# Treatment with direct oral anticoagulants or warfarin and the risk for incident diabetes among patients with atrial fibrillation: a population‐based cohort study

**DOI:** 10.1186/s12933-021-01263-0

**Published:** 2021-03-25

**Authors:** Ching-Lung Cheung, Chor-Wing Sing, Wallis C. Y. Lau, Gloria H. Y. Li, Gregory Y. H. Lip, Kathryn C. B. Tan, Bernard M. Y. Cheung, Esther W. Y. Chan, Ian C. K. Wong

**Affiliations:** 1 Department of Pharmacology and Pharmacy, Li Ka Shing Faculty of Medicine, The University of Hong Kong, Pokfulam, Hong Kong, China; 2 Department of Medicine, Li Ka Shing Faculty of Medicine, The University of Hong Kong, Hong Kong, China; 3 Research Department of Practice and Policy, School of Pharmacy, University College London, London, UK; 4 Department of Health Technology and Informatics, The Hong Kong Polytechnic University, Hong Kong, China; 5 Liverpool Centre for Cardiovascular Science, University of Liverpool and Liverpool Heart & Chest Hospital, Liverpool, UK; 6 Liverpool Health Partners, Liverpool, UK

**Keywords:** Diabetes, Atrial fibrillation, Anti-coagulant, Pharmacoepidemiology, DOAC, Warfarin, Association, Epidemiology

## Abstract

**Background:**

Diabetes mellitus is a common comorbidity of atrial fibrillation (AF), which can complicate the management of AF. The pharmacology of oral anticoagulants (OACs) have been implicated in pathogenesis of diabetes, but the relationship between different OACs and risk of diabetes remains unexamined. This study aimed to evaluate the risk of diabetes with use of different OACs in AF patients.

**Methods:**

Population-based retrospective cohort study using an electronic healthcare database managed by the Hong Kong Hospital Authority. Patients newly diagnosed with AF from 2014 through 2018 and prescribed OACs were included and followed till December 31, 2019. Inverse probability of treatment weighting based on the propensity score (PS) is used to address potential bias due to nonrandomized allocation of treatment. The risks ofdiabetes were compared between different new OAC users using propensity score-weighted cumulative incidence differences (CID).

**Results:**

There were 13,688 new users of OACs (warfarin: n = 3454; apixaban: n = 3335; dabigatran: n = 4210; rivaroxaban: n = 2689). The mean age was 75.0 (SD, 11.2), and 6,550 (47.9%) were women. After a median follow-up of 0.93 years (interquartile range, 0.21–1.92 years), 698 incident diabetes cases were observed. In Cox-regression analysis, dabigatran use was significantly associated with reduced risk of diabetes when compared with warfarin use [HR 0.69 (95% CI 0.56–0.86; P < 0.001)], with statistically insignificant associations observed for use of apixaban and rivaroxaban. The corresponding adjusted CIDs at 2 years after treatment with apixaban, dabigatran, and rivaroxaban users when compared with warfarin were − 2.06% (95% CI − 4.08 to 0.16%); − 3.06% (95% CI − 4.79 to − 1.15%); and − 1.8% (− 3.62 to 0.23%). In head-to-head comparisons between women DOAC users, dabigatran was also associated with a lower risk of diabetes when compared with apixaban and rivaroxaban.

**Conclusions:**

Among adults with AF receiving OACs, the use of dabigatran had the lowest risk of diabetes when compared with warfarin use.

**Supplementary Information:**

The online version contains supplementary material available at 10.1186/s12933-021-01263-0.

## Background

Atrial fibrillation (AF) is a prevalent condition worldwide and a leading cause of stroke, with oral anticoagulants as the primary preventive therapy for stroke among AF patients. Warfarin is a traditional anticoagulant which reduces blood coagulation by antagonizing the effect of vitamin K. Direct oral anticoagulants (DOACs) have been introduced as an alternative to warfarin and reducing blood coagulation by direct inhibition of blood coagulation factors, such as thrombin (dabigatran) and factor Xa (rivaroxban and apixaban), thus it is considered vitamin K-independent. Although DOACs are more convenient, safer, and effective drugs in stroke prevention, warfarin remains the most widely prescribed anticoagulant worldwide. Detailed investigation of the safety profile of warfarin is of clinical and public health importance.

Vitamin K has been shown to be important in glycemic control [1]. Clinical trials have demonstrated that vitamin K supplementation improved insulin sensitivity index (ISI) [2, 3], disposition index [2], reduced insulin resistance [4], 2-h post oral glucose tolerance test (OGTT) glucose [3, 5], and 2-h post OGTT insulin [3, 5]. Moreover, a Mendelian Randomization study suggests that higher circulating level of phylloquinone (dietary vitamin K1) may be causally associated with lower risk of type 2 diabetes [6]. Given the vitamin K antagonizing effect, we hypothesized that warfarin use may be associated with increased risk of diabetes. In addition, since diabetes is a common comorbidity that can complicate the management of AF [7], understanding the effects of different oral anticoagulants (OACs) on risk of diabetes is important. However, their relationship remains unexamined. Thus, the aim of this population-based cohort study was to determine the associations of different OACs with incident diabetes in patients with AF.

## Methods

### Data source

We collected anonymised electronic medical records from the Clinical Data Analysis and Reporting System (CDARS) managed by the Hong Kong Hospital Authority (HA), a statutory body that manages all public hospitals and clinics in Hong Kong [8]. Hong Kong HA serves a population of over 7.4 million and covers approximately 80% of hospital admissions in Hong Kong [9]. Clinical data including demographics, date and cause of death, hospital admission, pharmacy dispensing records, diagnosis, procedures, and laboratory test results are recorded and centralized in CDARS for research and audit. CDARS has been extensively used for conducting high quality real-world studies [10–13]. Details of CDARS have been discussed elsewhere [12].

### Study cohort

The study cohort included patients who were 18 years and above, had a first-ever diagnosis of AF (*International Classification of Disease, Ninth Revision, Clinical Modification [ICD-9-CM]* code 427.3) between January 1, 2014 and December 31, 2018, and subsequently received a prescription of OACs of interest before December 31, 2019. Although apixaban has been available for prescription in HA hospitals since 2013, only < 10 patients were prescribed apixaban in the year of 2013. To avoid bias due to unmatched year of prescription, the year of 2014 was selected as the commencement year of the study. Patients were excluded if they (i) had recorded diagnosis of valvular heart disease or hyperthyroidism; (ii) had transient AF defined as undergoing cardiac surgery or being diagnosed with myocarditis, pericarditis, or pulmonary embolism within 90 days prior to their first AF occurrence; (iii) had missing date of birth or sex information; or (iv) died during the first AF episode. The ICD-9-CM codes for the diagnosis were presented in the Additional file 1: Table S1. Clinical data of the study cohort were collected until December 31, 2019.

### Exposure and outcome

The outcome of interest was a new prescription of apixaban, dabigatran, rivaroxaban or warfarin after the AF occurrence. The date of first prescription of OACs after AF was defined as the index date. To identify new users, we excluded patients who had prescription of any OACs (apixaban, dabigatran, rivaroxaban, warfarin, or edoxaban) within 180 days prior to the index date. In addition, we excluded patients exposed to more than one OAC on the index date. Edoxaban was not included as one of the exposures due to the limited sample size and hence statistical power.

The outcome of interest was incident diabetes. We defined diabetes as a recorded diagnosis coded with ICD-9-CM 250.xx or a prescription of anti-diabetic medication. Although 250.xx included both type 1 and type 2 diabetes, the study cohort had a mean age > 70, hence it is expected that most, if not all, of the incident diabetes should be type 2 diabetes. To identify incident diabetes, patients who had a recorded diagnosis of diabetes on/before the index date or had a prescription of anti-diabetic medication within 1 year on or prior to the index date were excluded. Coding of diabetes has been previously validated [14].

### Inverse probability of treatment weighting (IPTW)

IPTW based on the propensity score (PS) is used to address potential bias due to nonrandomized allocation of treatment [15]. PS weight is used to create a pseudo-population so that the distribution of baseline characteristics between treatment groups are similar, thereby minimizing confounding bias. The PS for multiple treatment groups was estimated using generalized boosted model with a maximum of 10,000 regression trees for optimal balance between treatment groups [16]. The weights were derived to obtain the estimates for the average treatment effect in the population. IPTW has been shown to be a promising approach with significantly reduced bias in evaluating multiple drugs when compared with PS matching [17].

### Propensity score for multiple treatment groups using generalized boosted model

We used the generalized boosted model (GBM) to estimate the propensity score weights for each treatment group of anticoagulants. GBM is a nonparametric machine-learning method which involves an iterative process to capture complex and nonlinear relationships between treatment assignment and patient characteristics [16]. The iterative estimation can be tuned to find the propensity score model with optimal balance between treatment groups. Previous simulation studies have shown that GBM provides more stable weights and better balance of covariates compared to parametric logistic regression models [18, 19].

We used the “twang” package in R to compute the GBM. We set a maximum of 10,000 iterations (or regression trees) with an iteration stopping point that minimizes the absolute standardized mean difference of the effect size (es mean). The average treatment effect (ATE) weights were used to estimate the treatment effects in the entire population. The ATE weights were regarded as propensity score weights to fit the weighted Cox proportional hazard regression model.

The predictor variables in the PS generation included sex, age on the index date, calendar year of the index date, dispensing Institution (classified into seven hospital clusters in Hong Kong Hospital Authority), medical history of congestive heart failure, ischemic stroke or transient ischemic attack, chronic obstructive pulmonary disease, liver disease, chronic kidney disease, osteoporosis, osteoporotic fractures, rheumatoid arthritis and other inflammatory polyarthropathies, and falls (ICD-9-CM for the diagnosis is listed in Additional file 1: Table S1). Other predictor variables were recent use of relevant medications within 90 days on or before the index date, including angiotensin-converting enzyme inhibitors and/or angiotensin II receptor blockers, beta blockers, proton pump inhibitors, antidepressants (selective serotonin reuptake inhibitors and/or tricyclic antidepressants), and systemic glucocorticoids.

Standardized differences were calculated to access the balance of variables in the treatment groups. For multiple treatment groups, the maximum pairwise standardized differences were selected for assessment. Proposed cut-offs for acceptable standardized differences have ranged from 0.1 to 0.25 [20]. In this study, variables with standardized difference > 0.2 after IPTW were adjusted in the subsequent regression model.

### Statistical analysis

We followed the patients for a maximum period of 2 years. The follow-up was censored at the date of occurrence of outcome, discontinuation of treatment (defined as > 14-day time frame between consecutive prescription refill), switching to other OACs, death, end of follow-up period, or end of data availability, whichever earlier. Weighted Cox proportional hazard regression model using IPTW as weights with a robust variance estimator was used to estimate the hazard ratios (HRs) and 95% confidence intervals (CIs) for the association of OACs with diabetes over the entire follow-up period. The model was adjusted for covariates that were not well balanced after IPTW. The proportional hazard assumption of the Cox model was tested to be valid.

Cumulative incidence difference (CID) of diabetes at 6, 12, 18, and 24 months since treatment were compared between treatment groups, using the weighted cox model adjusted for non-balanced variables after IPTW [21, 22]. The 95% CIs for CID were estimated using bootstrap methods. We estimated the adjusted CID using the method proposed by P.C. Austin [21]. In brief, we used the data of the study cohort to fit a weighted Cox proportional hazard model adjusted for covariates that were not well-balanced after IPTW. Using the fitted model, we predicted the survival probability over 6, 12, 18, 24 months of follow-up periods for every patient. At each time point, we assigned all patients to the same treatment group, which is one of the four anticoagulant treatments (warfarin, apixaban, dabigatran, and rivaroxaban). The predicted survival probabilities were then averaged across the patients and the cumulative incidence can be obtained by (1-mean survival probability). The calculations were then repeated for each treatment. Finally, we calculated the differences in the adjusted cumulative incidence between treatment groups.

The 95% confidence interval (CI) for CID was estimated using bootstrap methods. Bootstrap methods allow for estimation of confidence intervals through the repeated sampling of data. Each bootstrap sample is randomly drawn with replacement from the original study cohort, such that the random sample has the same size as the original cohort. In this study, we used 500 bootstrap samples to generate the sampling distribution for the CIDs. The 95% CI for CID were obtained by the percentile method, which is the 2.5th and 97.5th percentiles in the sampling distribution of CID.

### Additional analyses

Subgroup analysis by sex and age group (< 65 and ≥ 65) was conducted. An interaction term was included in the Cox model to evaluate the interaction effects with sex and age. Since use of DOAC is more preferable than warfarin in patients with chronic kidney diseases at stages 1–3 [23], sensitivity analysis with exclusion of patients with chronic kidney diseases was conducted to reduce any unmeasured confounding. On the other hand, patients could have undiagnosed diabetes at baseline. To reduce bias due to misclassification of outcome, sensitivity analysis with exclusion of patients diagnosed with diabetes during the first 30 days of the follow-up period was performed. In addition, we computed the E-value of HRs to further assess the potential impact of any unmeasured confounding on our study. The E-value is defined as the minimum strength of association that an unmeasured confounder would need to have with both treatment and outcome, conditional on the measured covariates, to explain away an observed association [24].

A two-sided P-value < 0.05 was considered as statistically significant. All statistical analyses were conducted using R (version 3.6.0). The packages “twang” and “survival” were employed for IPTW and Cox regression analysis, respectively.

## Results

### Characteristics of study cohort

**Fig. 1:**
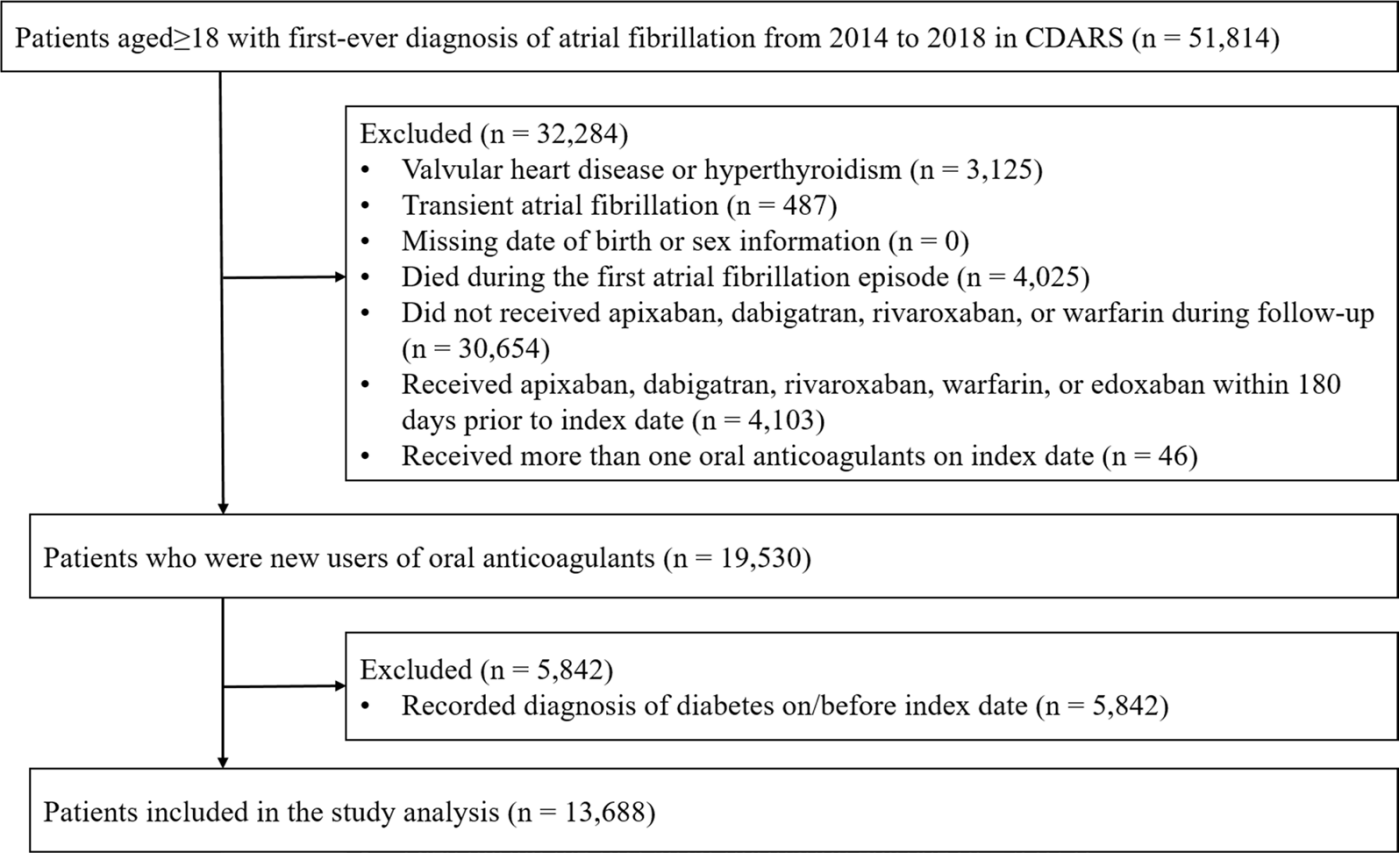
Screening flow chart of the study cohort

We identified 51,814 patients aged ≥ 18 with first-ever AF from 2014 through 2018 in CDARS. After excluding the ineligible patients (Fig. 1), there were 13,688 new users of OACs (warfarin [n = 3454]; apixaban [n = 3335]; dabigatran [n = 4210]; rivaroxaban [n = 2689]) were included in the analysis. Overall, the mean age was 75.0 ± 11.2, ranging from 72.9 ± 12.2 (warfarin) to 78.1 ± 10.8 (apixaban). The medium follow-up time was 339 days (interquartile range, IQR 77–701), ranging from 223 days (IQR 36–704) in warfarin users to 363 days (IQR 106–648) in apixaban users. All variables had a standardized difference of < 0.2, meaning that the IPTW weighted cohort were well-balanced. (Table 1). Thus, no variable was further adjusted in the subsequent analyses.

**Table 1:** Characteristics of study cohort

	Warfarin	Apixaban	Dabigatran	Rivaroxaban	Maximum pairwise standardized difference	
					Unweighted	Weighted
Subjects, n	3454	3335	4210	2689		
Age at index date, mean (sd)	72.9 (12.2)	78.1 (10.8)	74.4 (10.3)	74.9 (10.8)	0.47	0.06
Female, n (%)	1551 (44.9)	1699 (50.9)	2018 (47.9)	1282 (47.7)	0.12	0.03
Calendar year of index date, n (%)						
2014	704 (20.4)	90 (2.7)	403 (9.6)	371 (13.8)	0.64	0.1
2015	727 (21.0)	256 (7.7)	578 (13.7)	525 (19.5)	0.39	0.05
2016	670 (19.4)	486 (14.6)	785 (18.6)	621 (23.1)	0.22	0.01
2017	630 (18.2)	886 (26.6)	1045 (24.8)	547 (20.3)	0.19	0.03
2018	628 (18.2)	1163 (34.9)	1174 (27.9)	524 (19.5)	0.37	0.04
2019	95 (2.8)	454 (13.6)	225 (5.3)	101 (3.8)	0.41	0.05
Dispensing Institution by district, n (%)						
Hong Kong East	365 (10.6)	413 (12.4)	454 (10.8)	336 (12.5)	0.06	0.02
Hong Kong West	321 (9.3)	490 (14.7)	302 (7.2)	317 (11.8)	0.24	0.03
Kowloon Central	661 (19.1)	592 (17.8)	904 (21.5)	395 (14.7)	0.17	0.02
Kowloon East	399 (11.6)	284 (8.5)	370 (8.8)	703 (26.1)	0.52	0.04
Kowloon West	582 (16.9)	554 (16.6)	777 (18.5)	393 (14.6)	0.1	0.02
New Territories East	625 (18.1)	689 (20.7)	722 (17.1)	333 (12.4)	0.22	0.03
New Territories West	501 (14.5)	313 ( 9.4)	681 (16.2)	212 (7.9)	0.26	0.04
Medical history, n (%)						
Congestive heart failure	741 (21.5)	636 (19.1)	576 (13.7)	413 (15.4)	0.21	0.02
Stroke	525 (15.2)	706 (21.2)	797 (18.9)	460 (17.1)	0.15	0.03
COPD	313 (9.1)	304 (9.1)	338 (8.0)	215 (8.0)	0.04	0.04
Fall	566 (16.4)	637 (19.1)	617 (14.7)	420 (15.6)	0.12	0.03
Fracture	251 (7.3)	299 ( 9.0)	282 (6.7)	214 (8.0)	0.09	0.02
Chronic liver disease	23 (0.7)	16 (0.5)	14 (0.3)	4 (0.1)	0.08	0.04
Osteoporosis	37 (1.1)	65 (1.9)	51 (1.2)	41 (1.5)	0.07	0.004
Rheumatoid arthritis	29 (0.8)	39 (1.2)	36 (0.9)	22 (0.8)	0.04	0.03
Chronic kidney disease	187 (5.4)	79 (2.4)	42 (1.0)	49 ( 1.8)	0.28	0.05
Medication record in the past 90 days, n (%)						
ACE inhibitors	1504 (43.5)	1455 (43.6)	1529 (36.3)	1088 (40.5)	0.15	0.02
Beta blockers	2019 (58.5)	1959 (58.7)	2490 (59.1)	1649 (61.3)	0.06	0.02
Proton pump inhibitors	1169 (33.8)	1396 (41.9)	1321 (31.4)	877 (32.6)	0.22	0.04
Systemic corticosteroids	377 (10.9)	324 ( 9.7)	341 (8.1)	222 (8.3)	0.1	0.05
Anti-depressants	162 (4.7)	202 (6.1)	199 (4.7)	128 (4.8)	0.06	0.004

COPD: Chronic Obstructive Pulmonary Disease

### Risk of diabetes mellitus

A total of 698 incident diabetes were identified in the cohort. The IPTW weighted incidence rate in warfarin users was the highest (6.4 per 100 person-years; n = 195), followed by rivaroxaban users (5.1 per 100 person-years; n = 134), apixaban users (4.9 per 100 person-years; n = 177), and dabigatran users (4.3 per 100 person-years; n = 192). The median time to event since treatment ranged from 126 days (IQR 16–378) in warfarin users to 199 days (IQR 36–412) in rivaroxaban users (Table 2). In sub-group analysis by sex, warfarin users consistently had the highest weighted incidence of diabetes and the shortest time to event since treatment in both women and men (Table 2).

**Table 2:** Incidence of diabetes in patients receiving oral anticoagulants after atrial fibrillation

Treatment	Patients, n	Median follow-up time (IQR), d	Event, n	Median time to event (IQR), d	Crude incidence per 100 Person-years	Weighted^a^ incidence per 100 person-years
All patients						
Warfarin	3454	222 (36–704)	195	126 (16–378)	6.3	6.4
Apixaban	3335	363 (106–648)	177	174 (43–364)	5.3	4.9
Dabigatran	4210	363 (84–700)	192	196 (26–413)	4.5	4.3
Rivaxaban	2689	392 (98–730)	134	199 (36–412)	4.6	5.1
Women						
Warfarin	1551	221 (35–702)	78	154 (31–412)	5.6	5.8
Apixaban	1699	372 (106–651)	91	198 (46–360)	5.3	5.6
Dabigatran	2018	364 (78–683)	84	222 (18–423)	4.2	3.9
Rivaxaban	1282	416 (92–730)	67	186 (34–334)	4.7	5.4
Men						
Warfarin	1903	224 (38–705)	117	90 (8–344)	6.9	7
Apixaban	1636	348 (106–645)	86	134 (37–372)	5.3	4.1
Dabigatran	2192	358 (88–714)	108	192 (46–390)	4.8	4.7
Rivaxaban	1407	378 (102–730)	67	276 (45–480)	4.5	4.7

^a^Inverse probability of treatment weighted

Over the follow-up period, dabigatran use was significantly associated with reduced risk of diabetes when compared to warfarin use (HR, 0.69 [95% CI 0.56–0.86]; Table 3). The residual bias analysis showed that the corresponding E-value of the result point estimate was 2.25 in an HR scale. On the other hand, use of apixaban and rivaroxaban were marginally associated with reduced risk of diabetes when compared to warfarin use with an HR of 0.79 (95% CI 0.62–1.02) and 0.82 (95% CI 0.64–1.04), respectively (Table 3). With reference to warfarin use, the adjusted CID at 2-year after treatment with apixaban, dabigatran, and rivaroxaban were − 2.06% (95% CI − 4.08 to 0.16%), − 3.06% (95% CI − 4.79 to − 1.15%), and − 1.8% (95% CI − 3.62 to 0.23%), respectively (Table 4). Given apixaban and rivaroxaban are both Xa inhibitors, the risk of diabetes among users of either apixaban or rivaroxaban were also examined. These were observed to have lower risk of diabetes when compared to warfarin users (HR, 0.80 [95% CI 0.66–0.99]; Table 3). The corresponding cumulative incidence curves are provided in Additional file 1: Fig. S1.

**Table 3:** Risk of diabetes in patients with atrial fibrillation receiving DOACs and warfarin

	All patients		Women		Men		P_interaction_
	Hazard ratio (95% CI)	P	Hazard ratio (95% CI)	P	Hazard ratio (95% CI)	P	
DOACs vs Warfarin (ref.)							
Apixaban vs Warfarin	0.79 (0.62–1.02)	0.07	1.02 (0.69–1.50)	0.92	0.62 (0.46–0.84)	0.002	0.046
Dabigatran vs Warfarin	0.69 (0.56–0.86)	< 0.001	0.69 (0.50–0.96)	0.03	0.70 (0.53–0.92)	0.01	0.99
Rivaroxaban vs Warfarin	0.82 (0.64–1.04)	0.1	0.98 (0.68–1.40)	0.9	0.70 (0.51–0.97)	0.03	0.18
Apixaban/ Rivaroxaban vs Warfarin	0.80 (0.66–0.99)	0.04	1.00 (0.73–1.37)	0.99	0.66 (0.51–0.86)	0.002	0.048
DOACs vs DOACs (ref.)							
Apixaban vs Dabigatran	1.14 (0.90–1.46)	0.28	1.47 (1.02–2.13)	0.04	0.89 (0.66–1.21)	0.46	0.04
Rivaroxaban vs Dabigatran	1.18 (0.93–1.50)	0.17	1.41 (1.00–1.99)	0.05	1.01 (0.73–1.39)	0.96	0.17
Rivaroxaban vs Apixaban	1.03 (0.79–1.35)	0.82	0.96 (0.64–1.43)	0.83	1.13 (0.80–1.60)	0.48	0.54
Apixaban/ Rivaroxaban vs Dabigatran	1.16 (0.95–1.42)	0.14	1.44 (1.07–1.94)	0.02	0.95 (0.73–1.24)	0.7	0.04

**Table 4:** Absolute difference in adjusted cumulative incidence of diabetes (95% CI)

	6 months	12 months	18 months	24 months
DOACs vs Warfarin (ref.)				
Apixaban vs Warfarin	− 0.79 (− 1.55 to 0.06)	− 1.21 (− 2.44 to 0.09)	− 1.66 (− 3.34 to 0.13)	− 2.06 (-4.08 to 0.16)
Dabigatran vs Warfarin	− 1.17 (− 1.86 to − 0.41)	− 1.80 (− 2.86 to − 0.65)	− 2.47 (− 3.93 to − 0.92)	− 3.06 (− 4.79 to − 1.15)
Rivaroxaban vs Warfarin	− 0.69 (− 1.42 to 0.09)	− 1.06 (− 2.14 to 0.13)	− 1.46 (− 2.93 to 0.19)	− 1.80 (− 3.62 to 0.23)
Apixaban/ Rivaroxaban vs Warfarin	− 0.74 (− 1.39 to − 0.05)	− 1.14 (− 2.10 to − 0.08)	− 1.56 (− 2.83 to − 0.11)	− 1.93 (− 3.62 to − 0.14)
DOACs vs DOACs (ref.)				
Apixaban vs Dabigatran	0.38 (− 0.38 to 1.13)	0.59 (− 0.59 to 1.69)	0.81 (− 0.79 to 2.34)	1.00 (− 0.97 to 2.86)
Rivaroxaban vs Dabigatran	0.48 (− 0.20 to 1.11)	0.74 (− 0.31 to 1.73)	1.01 (− 0.43 to 2.31)	1.26 (− 0.52 to 2.86)
Rivaroxaban vs Apixaban	0.10 (− 0.73 to 0.85)	0.15 (− 1.07 to 1.34)	0.21 (− 1.49 to 1.85)	0.26 (− 1.81 to 2.30)
Apixaban/ Rivaroxaban vs Dabigatran	0.43 (− 0.14 to 0.93)	0.66 (− 0.22 to 1.42)	0.91 (− 0.31 to 2.00)	1.13 (− 0.38 to 2.47)

95% confidence interval was estimated using bootstrap methods

In head-to-head comparisons between DOACs, higher risks of diabetes were observed in users of apixaban (HR, 1.14 [95% CI 0.90–1.46]) and rivaroxaban (HR, 1.18 [95% CI 0.93–1.50]) separately, compared to dabigatran users, but the differences in risk were not statistically significant (Table 3). Users of either apixaban or rivaroxaban did not have a significantly increased risk of diabetes when compared to dabigatran users (HR, 1.16 [95% CI 0.95–1.42]; Table 3). In comparing the use of rivaroxaban with reference to apixaban, no significant association was observed for the risk of diabetes (Table 3). The corresponding cumulative incidence curves are provided in Additional file 1: Fig. S2.

### Subgroup analyses

In evaluating the association of different DOACs with diabetic risk with reference to warfarin use, there was a significant interaction between sex and effect of apixaban (P-interaction, 0.046; Table 3). Compared to warfarin use, apixaban use was significantly associated with decreased risk of diabetes in men (HR, 0.62 [95% CI 0.46–0.84]; E-value, 2.61) but not in women (HR, 1.02 [95% CI 0.69–1.50]). Similarly, use of rivaroxaban compared to warfarin was significantly associated with reduced risk of diabetes in men (HR, 0.70 [95% CI 0.51–0.97]; E-value, 2.2) but not in women (HR, 0.98 [95% CI 0.68–1.40]) (Table 3). There was also significant difference in the risk of diabetes between female and male users of either apixaban or rivaroxaban (P-interaction, 0.048), with male users significantly associated with lower risk of diabetes (HR, 0.66 [95% CI 0.51–0.86]) but not in female users (HR, 1 [95% CI 0.73–1.37]) (Table 3).

In head-to-head comparisons between DOACs, significant interaction with sex (P-interaction, 0.04) was observed in the comparison involving apixaban. In comparison with dabigatran use, apixaban use was significantly associated with increased risk of diabetes in women (HR, 1.47 [95% CI 1.02–2.13]; E-value, 2.31) but not in men (HR, 0.89 [95% CI 0.66–1.21]) (Table 3). Consistently, with reference to dabigatran users, a significant difference was observed between female and male users of either apixaban or rivaroxaban (P-interaction, 0.04), with the female users significantly associated with elevated risk of diabetes (HR, 1.44 [95% CI 1.07–1.94]) and male users had a null association with diabetic risk (HR, 0.95 [95% CI 0.73–1.24]; Table 3).

In all head-to-head comparisons, there was no significant interaction between age and effects of any DOACs on diabetic risk (P-interaction > 0.05 for all; Additional file 1: Table S2).

### Sensitivity analyses

Excluding patients with CKD (Additional file 1: Table S3) or undiagnosed diabetes (Additional file 1: Table S4) did not materially alter the significant associations observed in our main analyses.

## Discussion

In this population-based real-world study in patients with AF, we found that DOAC use overall was associated with reduced risk of diabetes, compared with warfarin. First, dabigatran use was significantly associated with a reduced risk of incident diabetes when compared with warfarin use. This association was robust in multiple sensitivity analyses. Second, although neither use of rivaroxaban nor apixaban was significantly associated with reduced risk of diabetes, a class effect (Xa inhibitor) was observed when users of rivaroxaban and apixaban were combined as a group for analysis. Third, there was a significant sex-specific association of apixaban and rivaroxaban. All DOACs indeed had a similar reduced risk of diabetes in men but not in women.

Our findings are partially in line with a recent study using Taiwan’s National Health Insurance Database, which showed DOAC was significantly associated with a lower risk of diabetes than warfarin [25]. However, the Taiwanese study showed that the significant association between use of DOACs and reduced risk of diabetes was only observed in women but not in men [25], whereas we observed that the potential protective effect of apixaban and rivaroxaban were only observed in men but not in women. On the other hand, they reported that the association of DOAC with incident diabetes had a significant interaction with age, whereas such age-interaction was absent in our findings. The discrepancies could be due to different sample demographics, database, and analytical approach. As the present study included a larger sample size with more events than the Taiwanese study, we have higher statistical power in detecting genuine association between drug use and risk of diabetes. The use of claim database in the Taiwanese study did not necessarily reflect clinical practice, whereas the clinical database used in the current study was derived from routine real-world clinical practice. The PS matching approach adopted in the previous study from Taiwan may lead to significant bias in multiple drug comparisons [17], and it can only be generalized to patients eligible to be included in specific drug-pair comparison. While the IPTW approach applied in the current study has been shown to be a promising approach in multiple drug comparisons with significant bias reduction [17], the findings generated from this weighted approach has a higher generalizability to the whole disease population. Moreover, there was no head-to-head comparison between DOACs in the previous Taiwanese study. Nevertheless, both studies do suggest that DOACs were significantly associated with reduced risk of diabetes, compared to warfarin.

Our findings are in line with previous studies that investigated the effects of dietary intake or supplementation of vitamin K on glucose homeostasis and reduced risk of diabetes. Although most, if not all, studies showed that vitamin K has no effect on fasting glucose, insulin, and glycated hemoglobin, more than one study indicated that vitamin K has a beneficial effect on ISI, 2-h OGTT glucose and insulin. The Framingham Offspring Study [26] showed that higher dietary intake of vitamin K was associated with lower 2-h OGTT insulin, 2-h OGTT glucose and improved ISI. Same findings were observed in clinical trials of vitamin K supplementation [2, 3, 5]. Similarly, risk of diabetes [27] and metabolic syndrome [28] was significantly lower among people with higher dietary intake of vitamin K. Given that the follow-up time was short in clinical trial, incident diabetes was not examined. Thus, our finding provides further evidence that antagonizing vitamin K using warfarin is associated with increased risk of diabetes. The relationship between vitamin K and risk of diabetes may be mediated by osteocalcin. Vitamin K is required in carboxylation of osteocalcin, which plays an important role in energy metabolism [29, 30]. Warfarin-treated patients had a significantly lower level of carboxylated osteocalcin [31], while carboxylated osteocalcin was potentially protective against diabetes and reported to be associated with reduced insulin resistance [32, 33], fasting glucose [33], and odds of metabolic syndrome [34] in human. Whether carboxylated osteocalcin plays a role in the risk of diabetes among AF patients prescribed different OACs requires further study.

Among the three DOAC evaluated, dabigatran was shown to be associated with the lowest risk of diabetes, while only marginal associations were observed for rivaroxaban and apixaban with a broadly similar HR observed (HR of 0.79 for apixaban vs. HR of 0.82 for rivaroxaban). Given that apixaban and rivaroxaban belong to the same class of drug (Xa inhibitor), users of these two DOACs were combined in one group in the analysis and a significant association was observed. This suggests the possibility of a class effect, especially when the estimates of these two drugs were highly similar. In addition, such marginal association observed for apixaban or rivaroxaban alone in the main analysis might be due to limited statistical power. Nevertheless, the difference in HR observed for use of apixaban and rivaroxaban versus dabigatran use, albeit statistically insignificant, could be due to sex-specific effect of apixaban and rivaroxaban.

We observed that the association of rivaroxaban and apixaban with risk of diabetes had a significant interaction with sex. In men, use of DOACs (apixaban, dabigatran, and rivaroxaban) were all significantly associated with reduced risk of diabetes when compared with warfarin use, with a HR ranging from 0.62 (apixaban) to 0.7 (dabigatran and rivaroxaban). However, the association in women was only observed in dabigatran users, while the HRs of apixaban (HR of 1.02) and rivaroxaban (HR of 0.98) users were close to 1 in men. This is indeed in agreement with two previous studies showing that rivaroxaban use reduced risk of stroke [35] and myocardial infarction [36] in men but not in women. However, our study differs from the Taiwanese study, which showed that DOAC was associated with reduced incident diabetes in women but not in men [25]*.* This discrepancy could be due to different sample demographics, database, and analytical approach. The mechanisms underlying these sex-specific association are unclear, but could be potentially due to the difference in sex hormones, sex-specific pathogenesis of diabetes [37], and/or other mechanisms.

Our study has important clinical implications. Diabetes is a common comorbidity of AF that complicates its clinical management. Presence of both diabetes and AF was associated with lower quality of life, higher risk of coronary events, heart failure, stroke, all-cause and cardiovascular mortality when compared to people with only one of the two diseases [38–40]. Based on the real-world clinical evidence, dabigatran use may lead to 3% reduction in absolute risk of diabetes at 2 years after treatment, when compared with use of warfarin. Meanwhile, use of apixaban or rivaroxaban may lead to 1.9% reduction in absolute risk of diabetes 2 years after treatment, when compared with warfarin use. If sex is taken into account, use of all the three DOACs were associated with reduced risk of diabetes in men, while dabigatran was the only DOACs which was also linked to decreased risk of diabetes in women. In addition to the present study, sex was reported to be a potential modifier of outcome in DOAC therapy [35, 36, 41]. Our findings do not only call for further investigation in the sex-specific effect of DOAC therapy on patients with AF, but also provide insights on the importance of sex-specific management of patients with AF in clinical practice, suggest that future risk of incident diabetes should be taken in account at initiation of OAC therapy to select the medication with minimal adverse effects.

### Strengths and limitations

There are several strengths in the current study. The study was conducted using a real-world clinical database with data of high quality. Given the real-world setting in nature, the generalizability is expected to be high. The association was robust in multiple sensitivity analyses. The sample size is large, thus providing ample power. The IPTW approach adopted in the current study has been shown to be a promising approach in performing multi-drugs comparison with significantly lower bias than the PS matching approach [17]. Nevertheless, there are limitations. First, this is an observational study, thus no causality can be established. Nevertheless, a well-conducted pharmacoepidemiology study with good-quality clinical data may improve internal validity of the findings. For example, our previous study showed that nitrogen-containing bisphosphonates was associated with reduced risk of myocardial infarction [42], and such finding was subsequently observed in a randomized controlled trial [43, 44]. Second, this is not a randomized study, thus residual confounding is possible. We therefore estimated the E-value to evaluate the effect of residual confounding in the current study. The E-values ranged from 2.2 to 2.6, indicating the unmeasured confounders that can explain away the current findings would need to be associated with both the use of DOACs and incident diabetes with a HR ranging from 2.2-fold to 2.6-fold, which is unlikely. Third, no fasting glucose or HbA1c data are available. However, the probability of presence of undiagnosed diabetes should be similar in all groups. Moreover, we addressed this issue by performing a sensitivity analysis, with exclusion of patients diagnosed with diabetes during the first 30 days after treatment, with the findings essentially unchanged (Additional file 1: Table S4). Fourth, measuring serum DOAC and warfarin may provide further insight on the relationship of DOAC and warfarin with incident diabetes, thus further study is warranted. Fifth, since this study was conducted in the Chinese, generalisability of the findings to other population is unknown.

## Conclusion

This study evaluated the effects of different OACs on risk of diabetes and demonstrated that dabigatran use had the lowest risk of incident diabetes when compared with use of warfarin, rivaroxaban, and apixaban. Rivaroxaban and apixaban may have a potential sex-specific effect on risk of diabetes.

## Supplementary Information

**Additional file 1: Table S1.** International Classification of Diseases, Ninth Revision, Clinical Modification (ICD-9-CM) codes used in the study. **Table S2.** Subgroup analysis by age group.** Table S3.** Sensitivity analysis excluding patients with chronic kidney diseases.** Table S4.** Sensitivity analysis by excluding patients diagnosed with diabetes during the first 30 days of the follow-up period.** Fig. S1.** Cumulative incidence of diabetes in patients receiving anticoagulants (warfarin, apixaban, dabigatran, and rivaroxaban) after atrial fibrillation.** Fig. S2**. Cumulative incidence of diabetes in patients receiving anticoagulants (warfarin, apixaban/rivaroxaban, and dabigatran) after atrial fibrillation.

## Data Availability

Data of this study are from at the HKHA of Hong Kong Special Administrative Region, and cannot be made publicly available.
