# Supplementary material for: Treatment with direct oral anticoagulants or warfarin and the risk for incident diabetes among patients with atrial fibrillation: a population‐based cohort study
Source: Cardiovasc Diabetol. 2021 Mar 25;20:71. doi: 10.1186/s12933-021-01263-0 (PMC7993481; doi:10.1186/s12933-021-01263-0)

Table S1. International Classification of Diseases, Ninth Revision, Clinical Modification (ICD-9-CM) codes used in the study

| **ICD-9-CM** | **Descriptions** |
| --- | --- |
| **Atrial fibrillation** | |
| 427.3 | Atrial fibrillation and flutter |
|  |  |
| **Valvular heart diseases or hyperthyroidism** | |
| 242 | Thyrotoxicosis with or without goitre |
| 394.0 | Mitral stenosis |
|  |  |
| Valvular heart surgery (procedure codes) | |
| 35.20 | Open and other replacement of unspecified heart valve |
| 35.22 | Open and other replacement of aortic valve |
| 35.24 | Open and other replacement of mitral valve |
| 35.26 | Open and other replacement of pulmonary valve |
| 35.28 | Open and other replacement of tricuspid valve |
|  |  |
| **Transient atrial fibrillation** | |
| Cardiac surgery (procedure codes) | |
| 00.5 | Other cardiovascular procedures |
| 35 | Operations on valves and septa of heart |
| 36 | Operations on vessels of heart |
| 37 | Other operations on heart and pericardium |
|  |  |
| Pericarditis | |
| 391 | Rheumatic fever with heart involvement |
| 393 | Chronic rheumatic pericarditis |
| 420 | Acute pericarditis |
| 423.2 | Constrictive pericarditis |
| 036.41 | Meningococcal pericarditis |
| 074.21 | Coxsackie pericarditis |
| 093.81 | Syphilitic pericarditis |
| 098.83 | Gonococcal pericarditis |
|  |  |
| Myocarditis | |
| 391.2 | Acute rheumatic myocarditis |
| 398.0 | Rheumatic myocarditis |
| 422 | Acute myocarditis |
| 429.0 | Myocarditis, unspecified |
| 032.82 | Diphtheritic myocarditis |
| 036.43 | Meningococcal myocarditis |
| 074.23 | Coxsackie myocarditis |
| 093.82 | Syphilitic myocarditis |
|  |  |
| Pulmonary embolism | |
| 415.1 | Pulmonary embolism and infarction |

Table S1. International Classification of Diseases, Ninth Revision, Clinical Modification (ICD-9-CM) codes used in the study (continued)

| **ICD-9-CM** | **Descriptions** |
| --- | --- |
| **Congestive Heart Failure** | |
| 398.91 | Rheumatic heart failure (congestive) |
| 402.01 | Malignant hypertensive heart disease with heart failure |
| 402.11 | Benign hypertensive heart disease with heart failure |
| 402.91 | Unspecified hypertensive heart disease with heart failure |
| 404.01 | Hypertensive heart and chronic kidney disease, malignant, with heart failure and with chronic kidney disease stage I through stage IV, or unspecified |
| 404.03 | Hypertensive heart and chronic kidney disease, malignant, with heart failure and with chronic kidney disease stage V or end stage renal disease |
| 404.11 | Hypertensive heart and chronic kidney disease, benign, with heart failure and with chronic kidney disease stage I through stage IV, or unspecified |
| 404.13 | Hypertensive heart and chronic kidney disease, benign, with heart failure and chronic kidney disease stage V or end stage renal disease |
| 404.91 | Hypertensive heart and chronic kidney disease, unspecified, with heart failure and with chronic kidney disease stage I through stage IV, or unspecified |
| 404.93 | Hypertensive heart and chronic kidney disease, unspecified, with heart failure and chronic kidney disease stage V or end stage renal disease |
| 428 | Heart failure |
|  |  |
| **Diabetes** |  |
| 250 | Diabetes mellitus |
|  |  |
| **Ischaemic stroke / Transient ischaemic stroke** | |
| 433.01 | Occlusion and stenosis of basilar artery with cerebral infarction |
| 433.11 | Occlusion and stenosis of carotid artery with cerebral infarction |
| 433.21 | Occlusion and stenosis of vertebral artery with cerebral infarction |
| 433.31 | Occlusion and stenosis of multiple and bilateral precerebral arteries with cerebral infarction |
| 433.81 | Occlusion and stenosis of other specified precerebral artery with cerebral infarction |
| 433.91 | Occlusion and stenosis of unspecified precerebral artery with cerebral infarction |
| 434 | Occlusion of cerebral arteries |
| 435 | Transient cerebral ischemia |
| 436 | Acute, but ill-defined, cerebrovascular disease |
| 437.0 | Cerebral atherosclerosis |
| 437.1 | Other generalized ischemic cerebrovascular disease |

Table S1. International Classification of Diseases, Ninth Revision, Clinical Modification (ICD-9-CM) codes used in the study (continued)

| **ICD-9-CM** | | **Descriptions** | |
| --- | --- | --- | --- |
| **Chronic Obstructive Pulmonary Disease** | | | |
| 490-496 | | Chronic Obstructive Pulmonary Disease and Allied Conditions | |
| 500 | | Coal workers' pneumoconiosis | |
| 501 | | Asbestosis | |
| 502 | | Pneumoconiosis due to other silica or silicates | |
| 503 | | Pneumoconiosis due to other inorganic dust | |
| 504 | | Pneumonopathy due to inhalation of other dust | |
| 505 | | Pneumoconiosis, unspecified | |
| 506.4 | | Respiratory conditions due to chemical fumes and vapors | |
|  | |  | |
| **Liver disease** | |  | |
| 456.0 | | Esophageal varices with bleeding | |
| 456.1 | | Esophageal varices without bleeding | |
| 456.2 | | Esophageal varices in diseases classified elsewhere | |
| 571.2 | | Alcoholic cirrhosis of liver | |
| 571.4 | | Chronic hepatitis | |
| 571.5 | | Cirrhosis of liver without mention of alcohol | |
| 571.6 | | Biliary cirrhosis | |
| 572.2 | | Hepatic encephalopathy | |
| 572.3 | | Portal hypertension | |
| 572.4 | | Hepatorenal syndrome | |
| 572.8 | | Other sequelae of chronic liver disease | |
|  | |  | |
| **Chronic Kidney Disease** | | | |
| 403 | | Hypertensive chronic kidney disease | |
| 404 | | Hypertensive heart and chronic kidney disease | |
| 582 | | Chronic glomerulonephritis | |
| 585 | | Chronic kidney disease (ckd) | |
| 590.0 | | Chronic pyelonephritis | |
|  | |  | |
| **Rheumatoid arthritis and other inflammatory polyarthropathies** | | | |
| 710.0 | | Systemic lupus erythematosus | |
| 710.1 | | Systemic sclerosis | |
| 710.4 | | Polymyositis | |
| 714.0 | | Rheumatoid arthritis | |
| 714.1 | | Felty's syndrome | |
| 714.2 | | Other rheumatoid arthritis with visceral or systemic involvement | |
| 714.81 | | Rheumatoid lung | |
| 725 | | Polymyalgia rheumatica | |
|  | |  | |
| **Osteoporosis** | | | |
| 733.0 | Osteoporosis | |  |

Table S1. International Classification of Diseases, Ninth Revision, Clinical Modification (ICD-9-CM) codes used in the study (continued)

| **ICD-9-CM** | | **Descriptions** | |
| --- | --- | --- | --- |
| **Fractures** | | |  |
| 805 | Fracture of vertebral column without mention of spinal cord injury | |  |
| 812 | Fracture of humerus | |  |
| 813 | Fracture of radius and ulna | |  |
| 814 | Fracture of carpal bone(s) | |  |
| 820 | Fracture of neck of femur | |  |
|  |  | |  |
| **History of falls** |  | |  |
| 781.2 | Abnormality of gait | |  |
| 781.3 | Lack of coordination | |  |
| 781.99 | Other symptoms involving nervous and musculoskeletal systems | |  |
| V15.88 | History of fall | |  |
| E880-E888 | Accidental Falls | |  |

Table S2. Subgroup analysis by age group

|  | All patients | |  | Age<65 | |  | Age≥65 | | P_interaction_ |
| --- | --- | --- | --- | --- | --- | --- | --- | --- | --- |
|  | Hazard ratio  (95% CI) | P |  | Hazard ratio  (95% CI) | P |  | Hazard ratio  (95% CI) | P |  |
| **DOACs vs Warfarin (ref.)** |  |  |  |  |  |  |  |  |  |
| Apixaban vs Warfarin | 0.79 (0.62-1.02) | 0.07 |  | 0.85 (0.44-1.64) | 0.63 |  | 0.78 (0.60-1.02) | 0.07 | 0.82 |
| Dabigatran vs Warfarin | 0.69 (0.56-0.86) | <0.001 |  | 0.90 (0.60-1.35) | 0.62 |  | 0.64 (0.50-0.82) | <0.001 | 0.16 |
| Rivaroxaban vs Warfarin | 0.82 (0.64-1.04) | 0.10 |  | 0.79 (0.47-1.31) | 0.35 |  | 0.83 (0.63-1.09) | 0.17 | 0.85 |
| Apixaban/Rivaroxaban vs Warfarin | 0.80 (0.66-0.99) | 0.04 |  | 0.82 (0.52-1.29) | 0.39 |  | 0.81 (0.64-1.01) | 0.06 | 0.97 |
| **DOACs vs DOACs (ref.)** |  |  |  |  |  |  |  |  |  |
| Apixaban vs Dabigatran | 1.14 (0.90-1.46) | 0.28 |  | 0.94 (0.49-1.82) | 0.86 |  | 1.22 (0.94-1.58) | 0.14 | 0.48 |
| Rivaroxaban vs Dabigatran | 1.18 (0.93-1.50) | 0.17 |  | 0.87 (0.52-1.45) | 0.59 |  | 1.29 (0.99-1.68) | 0.06 | 0.18 |
| Rivaroxaban vs Apixaban | 1.03 (0.79-1.35) | 0.82 |  | 0.92 (0.44-1.91) | 0.83 |  | 1.06 (0.80-1.41) | 0.70 | 0.73 |
| Apixaban/Rivaroxaban vs Dabigatran | 1.16 (0.95-1.42) | 0.14 |  | 0.90 (0.57-1.44) | 0.67 |  | 1.25 (1.00-1.56) | 0.05 | 0.21 |

Table S3. Sensitivity analysis excluding patients with chronic kidney diseases

|  | All patients | |  | Women | |  | Men | |
| --- | --- | --- | --- | --- | --- | --- | --- | --- |
|  | Hazard ratio  (95% CI) | P |  | Hazard ratio  (95% CI) | P |  | Hazard ratio  (95% CI) | P |
| **DOACs vs Warfarin (ref.)** |  |  |  |  |  |  |  |  |
| Apixaban vs Warfarin | 0.82 (0.63-1.05) | 0.12 |  | 1.08 (0.73-1.60) | 0.71 |  | 0.62 (0.46-0.85) | 0.003 |
| Dabigatran vs Warfarin | 0.72 (0.58-0.89) | 0.002 |  | 0.73 (0.52-1.02) | 0.07 |  | 0.71 (0.54-0.94) | 0.02 |
| Rivaroxaban vs Warfarin | 0.83 (0.65-1.06) | 0.14 |  | 0.99 (0.68-1.44) | 0.97 |  | 0.71 (0.51-0.99) | 0.04 |
| Apixaban/Rivaroxaban vs Warfarin | 0.82 (0.67-1.02) | 0.07 |  | 1.04 (0.75-1.44) | 0.83 |  | 0.67 (0.51-0.87) | 0.003 |
| **DOACs vs DOACs (ref.)** |  |  |  |  |  |  |  |  |
| Apixaban vs Dabigatran | 1.14 (0.89-1.46) | 0.29 |  | 1.48 (1.02-2.14) | 0.04 |  | 0.88 (0.64-1.19) | 0.40 |
| Rivaroxaban vs Dabigatran | 1.16 (0.91-1.47) | 0.22 |  | 1.36 (0.96-1.94) | 0.08 |  | 1.00 (0.73-1.39) | 0.99 |
| Rivaroxaban vs Apixaban | 1.02 (0.77-1.34) | 0.91 |  | 0.92 (0.62-1.38) | 0.70 |  | 1.14 (0.80-1.63) | 0.45 |
| Apixaban/Rivaroxaban vs Dabigatran | 1.15 (0.94-1.41) | 0.17 |  | 1.42 (1.05-1.92) | 0.02 |  | 0.94 (0.72-1.23) | 0.65 |

Table S4. Sensitivity analysis by excluding patients diagnosed with diabetes during the first 30 days of the follow-up period

|  | All patients | |  | Women | |  | Men | |
| --- | --- | --- | --- | --- | --- | --- | --- | --- |
|  | Hazard ratio  (95% CI) | P |  | Hazard ratio  (95% CI) | P |  | Hazard ratio  (95% CI) | P |
| **DOACs vs Warfarin (ref.)** |  |  |  |  |  |  |  |  |
| Apixaban vs Warfarin | 0.84 (0.64-1.11) | 0.21 |  | 0.97 (0.64-1.47) | 0.89 |  | 0.72 (0.50-1.03) | 0.07 |
| Dabigatran vs Warfarin | 0.73 (0.57-0.94) | 0.02 |  | 0.65 (0.44-0.95) | 0.03 |  | 0.81 (0.58-1.13) | 0.21 |
| Rivaroxaban vs Warfarin | 0.93 (0.70-1.23) | 0.6 |  | 0.97 (0.64-1.46) | 0.87 |  | 0.89 (0.61-1.31) | 0.56 |
| Apixaban/Rivaroxaban vs Warfarin | 0.88 (0.70-1.12) | 0.3 |  | 0.97 (0.68-1.38) | 0.86 |  | 0.81 (0.59-1.10) | 0.18 |
| **DOACs vs DOACs (ref.)** |  |  |  |  |  |  |  |  |
| Apixaban vs Dabigatran | 1.15 (0.88-1.50) | 0.32 |  | 1.50 (1.01-2.23) | 0.05 |  | 0.89 (0.63-1.25) | 0.49 |
| Rivaroxaban vs Dabigatran | 1.27 (0.97-1.66) | 0.08 |  | 1.49 (1.00-2.22) | 0.05 |  | 1.11 (0.77-1.60) | 0.58 |
| Rivaroxaban vs Apixaban | 1.11 (0.83-1.48) | 0.49 |  | 0.99 (0.65-1.52) | 0.98 |  | 1.25 (0.85-1.85) | 0.26 |
| Apixaban/Rivaroxaban vs Dabigatran | 1.21 (0.96-1.51) | 0.1 |  | 1.49 (1.07-2.09) | 0.02 |  | 1.00 (0.74-1.35) | 0.99 |

Fig. S1. Cumulative incidence of diabetes in patients receiving anticoagulants (warfarin, apixaban, dabigatran, and rivaroxaban) after atrial fibrillation.


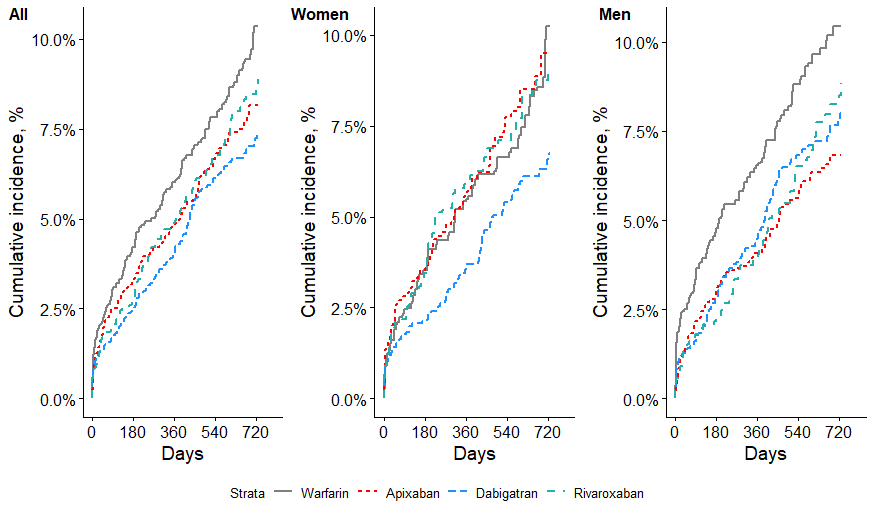


Fig. S2. Cumulative incidence of diabetes in patients receiving anticoagulants (warfarin, apixaban/rivaroxaban, and dabigatran) after atrial fibrillation.


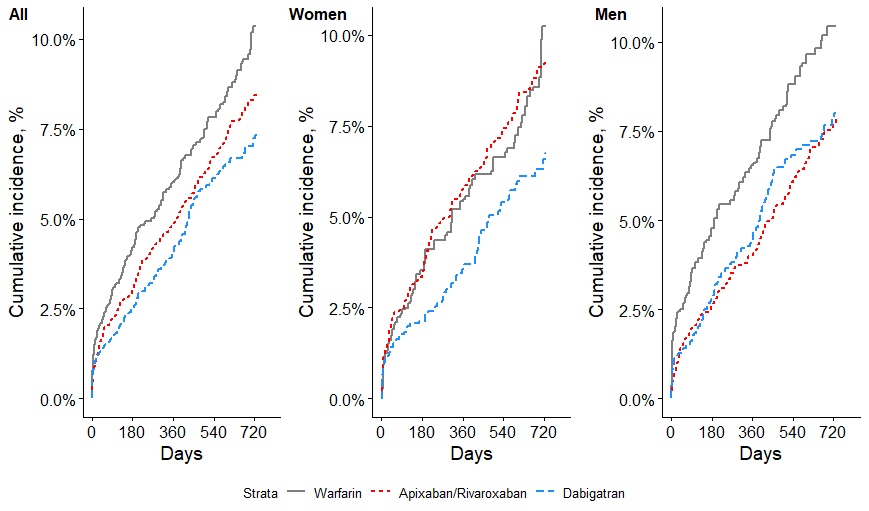

Supplement: Supplementary file 1 — Additional file 1: Table S1. International Classification of Diseases, Ninth Revision, Clinical Modification (ICD-9-CM) codes used in the study. Table S2. Subgroup analysis by age group. Table S3. Sensitivity analysis excluding patients with chronic kidney diseases. Table S4. Sensitivity analysis by excluding patients diagnosed with diabetes during the first 30 days of the follow-up period. Fig. S1. Cumulative incidence of diabetes in patients receiving anticoagulants (warfarin, apixaban, dabigatran, and rivaroxaban) after atrial fibrillation. Fig. S2. Cumulative incidence of diabetes in patients receiving anticoagulants (warfarin, apixaban/rivaroxaban, and dabigatran) after atrial fibrillation. [file 12933_2021_1263_MOESM1_ESM.docx]
